# Implementation of binarized neural networks immune to device variation and voltage drop employing resistive random access memory bridges and capacitive neurons

**DOI:** 10.1038/s44172-024-00226-z

**Published:** 2024-06-18

**Authors:** Mona Ezzadeen, Atreya Majumdar, Olivier Valorge, Niccolo Castellani, Valentin Gherman, Guillaume Regis, Bastien Giraud, Jean-Philippe Noel, Valentina Meli, Marc Bocquet, Francois Andrieu, Damien Querlioz, Jean-Michel Portal

**Affiliations:** 1 Univ. Grenoble Alpes, CEA, List, F-38000 Grenoble, France; 2 Univ. Grenoble Alpes, CEA, Leti, F-38000 Grenoble, France; 3 Aix-Marseille Univ., CNRS, IM2NP, Marseille, France; 4 Université Paris-Saclay, CNRS, Centre de Nanosciences et de Nanotechnologies, 91120 Palaiseau, France

**Keywords:** Electrical and electronic engineering, Electronic devices, Computational nanotechnology

## Abstract

Resistive Random Access Memories (ReRAM) arrays provides a promising basement to deploy neural network accelerators based on near or in memory computing. However most popular accelerators rely on Ohm’s and Kirchhoff’s laws to achieve multiply and accumulate, and thus are prone to ReRAM variability and voltage drop in the memory array, and thus need sophisticated readout circuits. Here we propose a robust binary neural network, based on fully differential capacitive neurons and ReRAM synapses, used in a resistive bridge fashion. We fabricated a network layer with up to 23 inputs that we extrapolated to large numbers of inputs through simulation. Defining proper programming and reading conditions, we demonstrate the high resilience of this solution with a minimal accuracy drop, compared to a software baseline, on image classification tasks. Moreover, our solution can achieve a peak energy efficiency, comparable with the state of the art, when projected to a 22 nanometer technology.

## Abstract (web summary)

Mona Ezzadeen and co-authors demonstrate a compute-in memory cell with a low consumed power per operation. In silicon implementation with 23 inputs is successfully used to solve benchmarking tasks of digit recognition.

## Introduction

The energy efficiency of artificial intelligence (AI) is strongly limited by data movement between processing cores and the various memories of the hierarchy^1^. Near and in-memory computing approaches constitute major leads to support AI algorithms efficiently, as these concepts drastically minimize data movement. Some of the most efficient realizations of these approaches implement Binarized Neural Networks (BNN), which use binarized weights and activations that simplify the computational process and alleviate memory usage while retaining high accuracy^2,3^.

In-memory neural network organization fits particularly well with arrays of resistive memories (ReRAMs), also called memristors^4^: rows represent input neurons, ReRAM cells map synaptic weights, and columns represent output neurons. Using such a topological implementation, the fundamental operation of neural networks, multiplication and accumulation (MAC), can be realized naturally using Ohm’s and Kirchoff’s laws. Unfortunately, even if this approach can, in principle, compute MACs with any number of inputs, it faces several challenges:

- ReRAM device variability has a strong impact on MAC accuracy in such analog approaches,
- applying Ohm’s and Kirchoff’s laws through multiple ReRAM cells in parallel results in current density issues with related voltage (IR) drop phenomenon.

For these reasons, in this work, we propose and demonstrate experimentally an alternative approach that remains analog but is immune to variability and IR drop effects. Our neuron circuit introduces a novel approach for XNOR operations, based on 2T2R cells used in a resistive divider fashion. The 2T2R nature of our cell strongly reduces the impact of variability. As the two ReRAM cells are connected in series, our approach always involves a high-resistance device in the resistor divider, limiting the read current and leading to a negligible IR drop effect. This in-series connection of the two cells differentiates our work from previous works using 2T2R bit cells^5,6^, and also allows us to rely on lightweight periphery circuitry: simple inverter gates are used to read weights, while simultaneously realizing the multiplication function of the neural network. Instead of using current Kirchoff’s law, following an approach initially proposed for SRAM^7,8^, accumulation and output neuron activation are obtained using capacitive divider bridges, which are more energy-efficient than equivalent digital circuits. To our knowledge, our work is the first to combine in-ReRAM XNOR operations with the use of a capacitive divider at the bottom of the ReRAM array to perform the popcount operation. We present an experimental validation of our concept on a test chip, manufactured in a hybrid CMOS/ReRAM 130-nanometer technology, and simulation-based predictions for a more advanced 22-nanometer node.

The advantage of our approach with regards to the SRAM-based pioneering work^7,8^ is two-fold. ReRAM cells are more compact than SRAM, bringing better scalability and lower cost. More profoundly, as ReRAM is non-volatile, the power supply can be turned off without losing the programmed neural network. This feature allows zero standby power and is particularly appropriate for embedded applications. ReRAM has demonstrated 10-years retention, even under challenging conditions^9^.

Our approach naturally offers a near-immunity to the effects of ReRAM variability and IR drop. Other fabricated in- and near-memory computing ReRAM circuits address these issues by using sophisticated readout circuitry and/or limiting the number of inputs of the in-memory MAC operations. These strategies, which our approach avoids, are problematic. Complex readout circuitry has a high energy and area cost. Limiting the number of in-memory MAC inputs means that only very partial MACs can be realized in-memory, and additional conventional digital circuitry (registers and adders) is necessary to compute final neuron activations. We now summarize the main techniques followed in the literature. Impressive realizations that compute 256^10^, 196^11^ and 784^12^-inputs MAC have been previously published. Each of these studies employed different methods to address ReRAM and periphery circuits non-idealities. For instance, model-driven chip calibration, noise-resilient neural-network training and analog weight programming, and chip-in-the-loop model fine-tuning can be used^10^. Complex periphery circuits can also compensates for the non-idealities, to implement a “max value search of MAC operation” strategy^11^, or a local current cancellation technique involving analog-to-digital and digital-to-analog conversion with numerous clock-cycles for proper conversion and multi-bits precision weights and activations^12^. Another approach consists of mitigating the challenges of in-memory computing by limiting the number of inputs of in-memory MAC operations^13–19^. In particular, a one-bit input, ternary-weighted, and three-bits output ReRAM-based MAC operations with nine up to 25 inputs has been demonstrated^13^. This work uses separate ReRAM arrays to store positive and negative weights, coupled with a complex analog sensing circuit to overcome the sense amplifier offsets and the small sensing margin due to the ReRAM variability. Another approach^14^ uses multiple ReRAM cells to store three-bit signed weights rather than multi-level cells and applies sequential word line pulses to implement two-bit inputs. A sophisticated analog sense amplifier then generates a three-bits MAC output value. Despite the analog circuit design complexity, and due to the ReRAM variability, this work used a maximal MAC size of nine inputs. It also shows a tradeoff between the CIFAR-10 accuracy and the achieved energy efficiency. To increase the MAC number of inputs while limiting the ReRAM read current and preserving inference accuracy, an alternative method^16^ consists of introducing input-aware multi-bit bit line clamping and source line biasing with a single activated word line, along with multiple current sensing optimizations. This approach is still limited to 16 inputs per MAC. To push the MAC precision up to eight-bit inputs and weights, an asymmetric group-modulated input scheme along with voltage-mode sense amplifiers was proposed^17^. However, the maximal number of inputs per MAC is only four. By proposing a direct-current-free time-space ReRAM-based MAC operation, another work^18^ achieved a high energy efficiency of 416.5 TOPS/W in the binary case. However, the MAC number of inputs is still low, limited to 16 accumulations. A ReRAM-based in-memory computing approach based on a voltage division mechanism on entire columns^19^ has also been published. It helps lowering the sensitivity to ReRAM variability, but without suppressing the need for important readout circuitry, and still being limited to small MAC sizes with a maximum of nine inputs.

Partial and preliminary results of this work have been published at a conference, based on simulations of our approach and measurements on a test die lacking the periphery circuits^20^. This version adds silicon-based results with a full BNN test-chip implementation and characterization, and a complete modeling of the neuron’s error probability with regard to the neuron size, operation voltages, clock frequencies, and programming conditions.

## Results and discussion

### RRAM-based capacitive neuron: overview and test chip experimental validation

#### Binarized neural network test chip

**Fig. 1:**
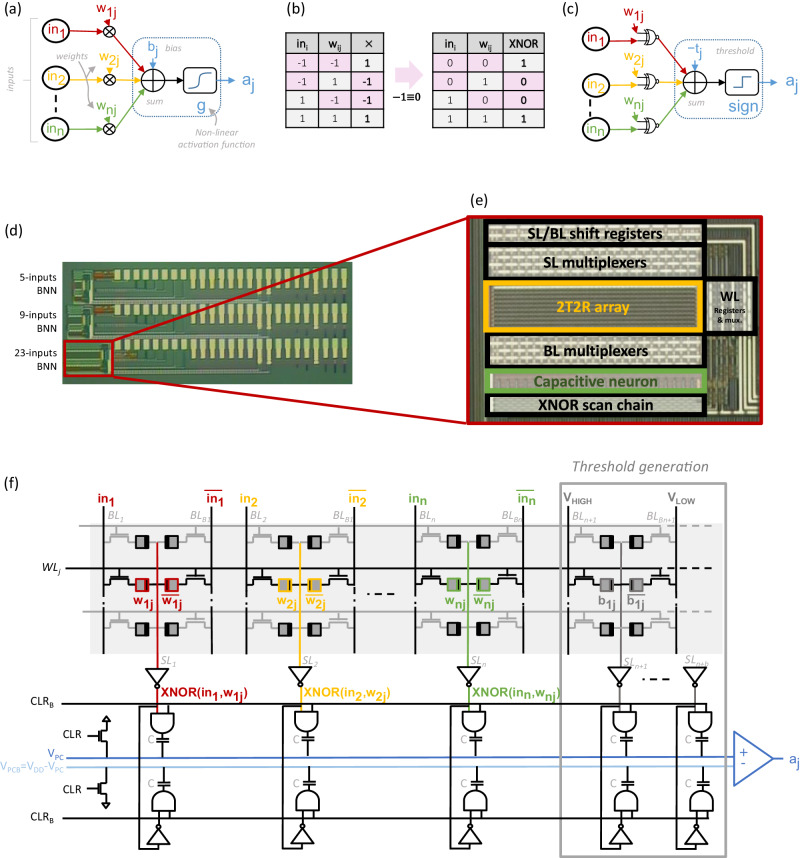
Binarized neural network test chip. **a** Neuron structure in a conventional full precision neural network with *n* activation inputs. **b** Multiplication and XNOR truth table equivalence for inputs and weights in {−1, 1}. The multiplication operation is equivalent to the eXclusive NOR (XNOR) operation when replacing −1 values by 0. **c** Same neuron structure as in (**a**), in the binary case. **d** Binary Neural Network (BNN) test chip optical microscopy photograph, with the three implemented neuron sizes (5,9 and 23 inputs). The test chip is designed and fabricated in a 130-nanometer Complementary Metal-Oxide Semiconductor (CMOS) technology with co-integrated Resistive Random Access Memory (ReRAM) cells in the back-end-of-line between metal layers four and five. **e** Detail of the 23-inputs neuron version. **f** Global architecture of the BNN circuit of the test chip, with 2-Transistors-2-Resistors (2T2R) ReRAM cell using complementary coding to achieve robust XNOR operation, and fully differential coding of popcount and threshold with capacitive bridge to enhance comparison margin. SL Source Line, BL Bit Line, WL Word Line.

Neural networks are composed of neurons, which receive *n* inputs *in*_*i*_ and produce a single output activation

1 $${a}_{j}=g\left({\sum}_{i}({w}_{ij}\times i{n}_{i})+{b}_{j}\right),$$

where *w*_*ij*_ are the synaptic weights connecting the neurons, *b*_*j*_ the neuron biases, and *g* a non-linear activation function (see Fig. 1a). In binarized neural networks, neuron activations and weights only take ± 1 values, greatly simplifying eq. (1). The multiplication operation between an input *in*_*i*_ and a weight *w*_*ij*_ becomes an XNOR operation (replacing -1 values by 0, see Fig. 1b), and the accumulation operation becomes a population count (popcount) operation. The non-linear activation function g is replaced by the sign function (Fig. 1c). The neuron output activation *a*_*j*_ thus becomes

2 $${a}_{j}={{{{{{{\rm{sign}}}}}}}}({{{{{{{{\rm{POPCOUNT}}}}}}}}}_{i}({{{{{{{\rm{XNOR}}}}}}}}({w}_{ij},i{n}_{i}))-{t}_{j}),$$

where *t*_*j*_ is a threshold value given by training.

Our test chip aims at computing this equation robustly and efficiently. It is designed and fabricated in a 130-nanometer CMOS technology with co-integrated ReRAM cells in the back-end-of-line between metal layers four and five (see the “Test chips fabrication" section in Methods). Our test chip includes three versions of the same BNN circuit, differing by their number of inputs: 5, 9, and 23 (Fig. 1d). The three versions implement a ReRAM array of size 10 × 5, 10 × 9 and 10 × 23 respectively. Figure 1e shows a micro-photography of the 23-inputs neuron circuit, which we use throughout this work. The core of the circuit is composed of a ReRAM array storing the weights and a capacitive neuron circuit at the bottom of the array. Additionally, shift registers control multiplexers that connect the desired SLs and BLs to metal pads, making ReRAM cells directly accessible for characterization purposes. For validation and error rate extraction, a scan chain captures the XNOR values in parallel and outputs them serially at the end of the output neuron operation. The capacitive bridge is designed with 105-femtofarad capacitors.

As illustrated in Fig. 1f and following eq. (2), our binarized neural network circuit is composed of

- 2T2R-based XNOR operators using a fully differential in-memory computing approach;
- two capacitive bridges connected respectively to the XNOR outputs and their complementary values, to implement the popcount operator in analog;
- extra bias capacitors on the two capacitive bridges to implement the threshold value;
- a near-memory comparator to perform the sign function and the difference between the popcount and the threshold values.

The output of the comparator is the binary value of the neuron activation.

We now describe these elements in detail.

#### RRAM-based robust in memory XNOR operation

**Fig. 2:**
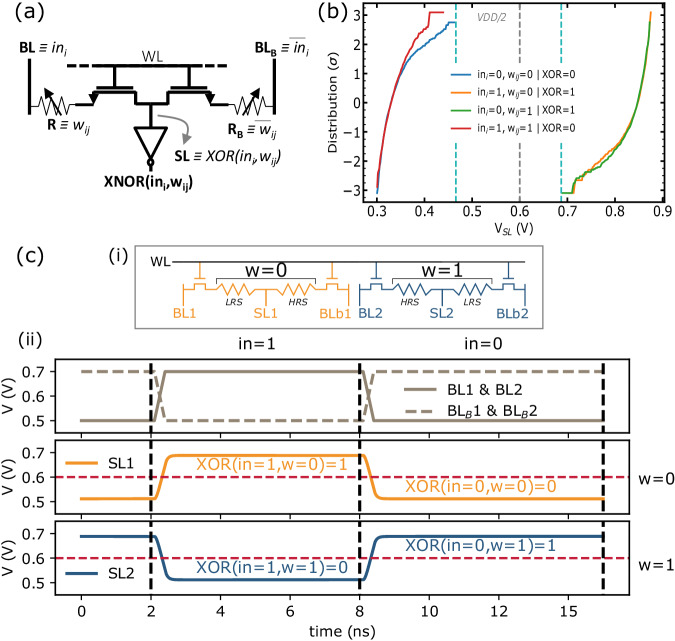
2-Transistors-2-Resistors (2T2R) bit cell with in-memory eXclusive NOR (XNOR) functionality. **a** Schematic of our proposed bit cell. The weights, stored in the Resistive Random Access Memory (ReRAM) 2-Transistors-2-Resistors (2T2R) cell, and the activation input, applied on the Bit Line (BL)/BL_B_, are both coded in a complementary fashion. This creates a voltage divider structure whose middle point is the Source Line (SL) and is the image of the eXclusive OR (XOR) operation between the weight and the activation input. The final XNOR value is generated by an inverter gate at the bottom of the SL. **b** XOR ( ≡ SL) measured distributions with V_LOW_ = 0.3 volts and V_HIGH_ = 0.9 volts on a 1204 2T2R ReRAM array programmed using I_CC_ = 200 microamperes and V_G_(reset) = 3.3 volts, V(reset) = 2.5 volts. **c** Simulated XOR operation on two 2T2R cells on adjacent columns selected simultaneously (c.i). The first cell stores a weight equal to zero (w = 0) while the second cell stores a weight equal to one (w = 1). A V_read_ of 0.2 V is used, corresponding to V_LOW_ = 0.5 volts and V_HIGH_ = 0.7 volts. The obtained plot (c.ii) illustrates successfully the complete XOR truth table. WL Word Line.

As depicted in Fig. 1f, the weights of a given neuron are stored in a single row of a ReRAM array. A key idea of our work is to rely on 2T2R (two transistors - two resistors) ReRAM cells (Fig. 2a) connected in series, forming a resistive bridge. Synaptic weights *w*_*ij*_ are coded in a complementary fashion in the two ReRAM of the 2T2R cells, meaning that depending on the value of the synaptic weight, either the left (R) or the right (R_B_) ReRAM cell is programmed to a High Resistance state. The complementary ReRAM is programmed to Low Resistance State (see Method for details on weight programming operations). The central point of the resistance bridge – the source line SL – is therefore pulled either toward the left or the right bit line, depending on the synaptic weight value. The input neuron values are presented on the two bit lines also in a complementary fashion, meaning that depending on the input neuron value, either the left or the right bit line is at the lowest voltage. The combination of these two effects means that the source line naturally follows an exclusive OR (XOR) between the weight and the neuron input (see Table 1): the memory array performs XOR operations directly within memory (the “In-memory XNOR operation” section in Methods describes this operation more mathematically). To illustrate this operation, we measured the source line voltage distributions for the four cases of the XOR truth table on an independent test chip featuring a 1024 2T2R ReRAM array without neuron circuits (see ref. ^20^), therefore allowing the direct measurement of the source line voltages. The results shown in Fig. 2b reveal significant read margins between the XOR distributions tails and V_DD_/2. Figure 2c demonstrates successfully the simulated XOR operation for the complete truth table. In our full system, these source line voltages are used as input to inverter gates at the bottom of each source line to compute the inverted XOR, namely XNOR, values.

**Table 1:** Truth table of the proposed in-memory XNOR operation

Weights			Neuron input			XOR (SL)		XNOR
bin	R	R_B_	bin	BL	BL_B_	(V)	bin	
0	LRS	HRS	0	V_LOW_	V_HIGH_	<V_DD_/2	0	1
0	LRS	HRS	1	V_HIGH_	V_LOW_	>V_DD_/2	1	0
1	HRS	LRS	0	V_LOW_	V_HIGH_	>V_DD_/2	1	0
1	HRS	LRS	1	V_HIGH_	V_LOW_	<V_DD_/2	0	1

The in-memory XNOR operation is expected to fail only if the device programmed into low-resistance state has a resistance higher than the device programmed into high resistance. This situation has a very low probability to appear, since both devices have to be programmed improperly. Additionally, the nonlinearity of the inverter amplifies the signal, leading to clean binary outputs. These two elements make our approach highly robust to variability. Note that a 2T2R strategy has already been proposed to reduce bit errors in a digital context^5,6^, with devices connected in parallel read by precharge sense amplifiers. A unique benefit of our approach is that, as the two devices are connected in series, the current paths in the memory array always include a high-resistance device. Therefore, the in-memory XNOR operation relies on a low current, regardless of the input and weight values. This also makes our approach naturally immune to IR-drop effects (see Method for IR-drop projection on large memory array).

#### Near-memory popcount and sign operation

Popcount and sign operations are performed near memory using a switched-capacitor addition circuit and a comparator (see Fig. 1f), following an approach inspired by an SRAM-based work^7,8^. The use of a switched-capacitor circuit is highly energy-efficient with regards to a digital implementation, as, unlike in in-memory MAC realizations exploiting Kirchoff’s current law, no direct current needs to be applied: energy is only consumed when the capacitors are switching. The popcount circuit is based on a fully differential approach with two capacitive bridges connected to complementary inputs. The “On-chip operation of the popcount computation” section in Methods lists the different steps of the popcount operation, realized in one clock cycle, and which leads the voltages of two capacitive bridges to

3 $${V}_{{{{{{{{\rm{PC}}}}}}}}}=\frac{m}{n}{V}_{{{{{{{{\rm{DD}}}}}}}}}$$

and

4 $${V}_{{{{{{{{\rm{PCB}}}}}}}}}={V}_{{{{{{{{\rm{DD}}}}}}}}}-\frac{m}{n}{V}_{{{{{{{{\rm{DD}}}}}}}}},$$

where *m* is the number of XNOR outputs equal to one, i.e., the popcount value, and *n* is the total number of XNOR outputs connected to each capacitive bridge. The comparator takes as input these two voltages and produces as output the binary activation *a*_j_ of the neuron. This means that the activation is set to one when more than half of the XNOR values are equal to one (i.e., $m \, > \, \frac{n}{2}$). Therefore, the circuit naturally implements a neuron (see equation (2)) with a threshold *t*_*j*_ of $\frac{n}{2}$.

Statistically, neural network inference simulations (see section “BNN circuit performances at neural network scale”) show that a threshold setting capability of ± 5% around the mean $\frac{n}{2}$ value is necessary and typically sufficient to achieve a good accuracy. To offer this capability, we added *b* = 2 × ⌊0.05 $*$ *n*⌋ capacitors to each bridge in a complementary fashion. These extra capacitors are connected to the source line of additional columns in the ReRAM array, where the threshold are programmed (see the “Threshold adjustment in the near-memory popcount operation” section in Methods for the details concerning the threshold circuit).

### Characterization results and error model

#### Characterization results on the BNN chips

**Fig. 3:**
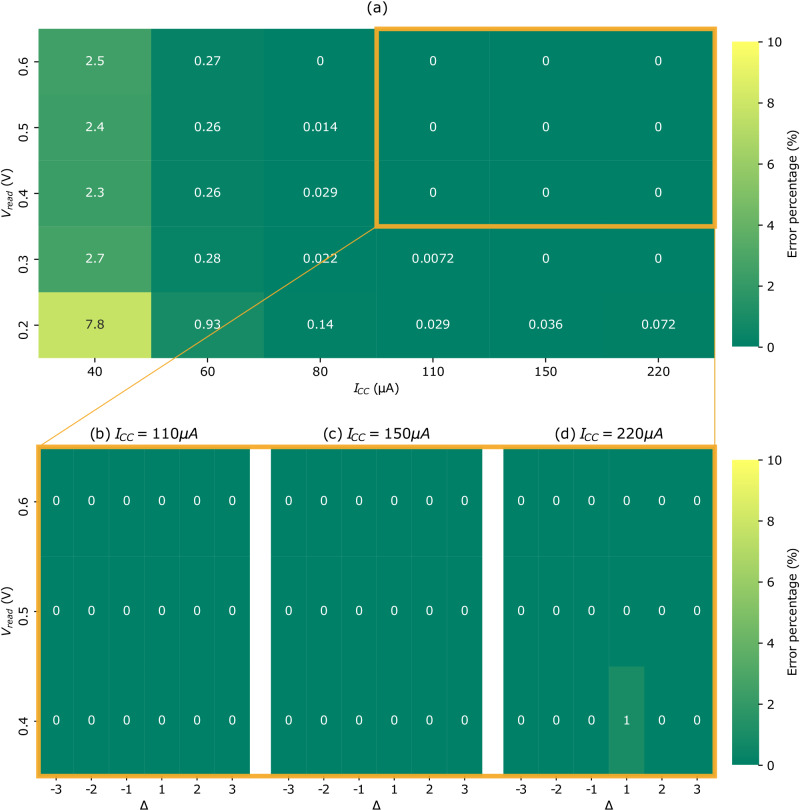
Experimental measurement of the error rate for eXclusive NOR (XNOR) and neuron activations. **a** Measured XNOR error percentages for different compliance current I_CC_ and read voltage V_read_. **b**–**d** Measured neuron error percentages for I_CC_ equal to 110 microamperes to 220 microamperes resp., for V_read_ ranging from 0.4 volts to 0.6 volts and for Δ - the difference between popcount and threshold values, here represented by the difference between the number of inputs set to one in the neuron’s two capacitive bridges - ranging from −3 to 3. A value of zero means that no error was measured. The experimental details for obtaining these results are presented in the “Test chip characterization" section in Methods.

To validate the functionality of our BNN circuit and its robustness against ReRAM variability, we first performed an extensive set of inference operations. These operations are performed on the 23-inputs circuit of Fig. 1, with read voltages V_read_ ranging from 0.2 to 0.6 volts and six different compliance currents used during weights programming. (The precise read voltage and compliance current definitions are given in the “Test chip characterization” section in Methods.) Fig. 3 shows the measured XNOR and neuron error probabilities (the methodology for obtaining these results is presented in the “Test chip characterization” section in Methods). We measured no errors for a compliance current greater or equal to 110 microamperes and a read voltage greater than 0.3 volts. Figure 3b–d shows that in these conditions, we also measured no errors in the neuron values, even for minimal value of the difference between popcount and threshold values Δ (here represented by the difference between the number of inputs set to one in the neuron’s two capacitive bridges). These results show that our circuit is capable of highly robust computation.

**Fig. 4:**
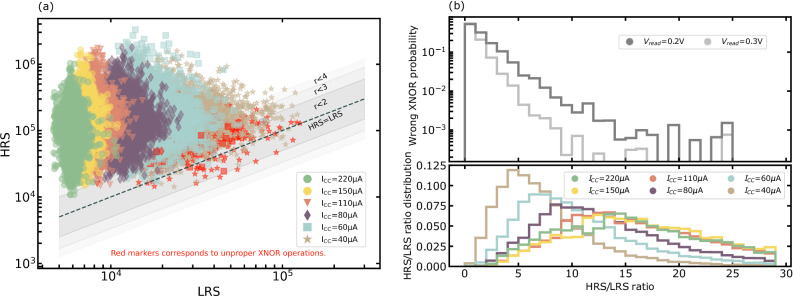
Statistical measurements of eXclusive NOR (XNOR) operation. **a** (Hight Resistive State (HRS), Low Resistive State (LRS)) scatter plot for the six considered I_CC_ values (13,800 points each) with V_read_ = 0.3 V. Red markers correspond to improper eXclusive NOR (XNOR) operations. The dotted line corresponds to the HRS = LRS boundary, and the gray zones correspond to different r=HRS/LRS ratios. **b** XNOR error probability with regards to HRS/LRS ratio, along with the HRS/LRS ratio distributions for the six considered I_CC_ values. The experimental details for obtaining these results are presented in the “Test chip characterization” section in Methods.

By contrast, we can see in Fig. 3a that errors start to occur for compliance currents lower than 110 microamperes or read voltages lower than 0.3 volts. To better understand these regimes, we programmed a total of 13,800 weights on the test chip, for each of the six considered programming conditions, and for all considered read voltages. We measured their respective High Resistive State (HRS) and Low Resistive State (LRS) values and the XNOR output. The experimental results for a read voltage of 0.3 volts are presented in the scatter plot in Fig. 4a. Red markers correspond to improper XNOR operations. The results confirm that the lower the compliance current, the wider the (HRS, LRS) scatter plot is. Couples close to the HRS=LRS diagonal present a low HRS to LRS ratio and, therefore, a significant probability of giving erroneous XNOR outputs, as shown in Fig. 4b. For instance, a ratio between four and six leads to an XNOR error rate of 0.9% for V_read_ = 0.3 volts. Figure 4b also shows that the effect is more substantial for lower read voltages: with V_read_ = 0.2 volts, an HRS/LRS ratio between four and six leads to an XNOR error rate of 3.9%.

#### Simulation results of the neuron circuit for scaled-up BNNs

In our fabricated circuits, we saw that the voltage difference between the two capacitive bridges remains sufficiently large to avoid any output neuron activation error, when XNOR outputs are error-free (Fig. 3b–d). However, such errors may appear in circuits with larger input numbers, for low Δ values, as this situation would lead to low voltage differences at the comparator inputs: voltages *V*_*PC*_ and *V*_*PCB*_ can become very close. We, therefore, performed extensive Monte Carlo simulations of circuits with BNN sizes up to 513 inputs neurons and clock periods ranging from 4 to 20 nanoseconds, for the full range of possible popcount and threshold combinations (Δ values). We consider global and local sources of variability, including mismatch, at three standard deviations. For all simulated cases, 1000 runs are performed. To include the ReRAM variability, the source line measured distributions of Fig. 2c, corresponding to a compliance current of 200 microamperes and a read voltage of 0.6 volts (and thus to error-free XNOR operations), are directly injected at the XNOR inverter’s inputs.

**Fig. 5:**
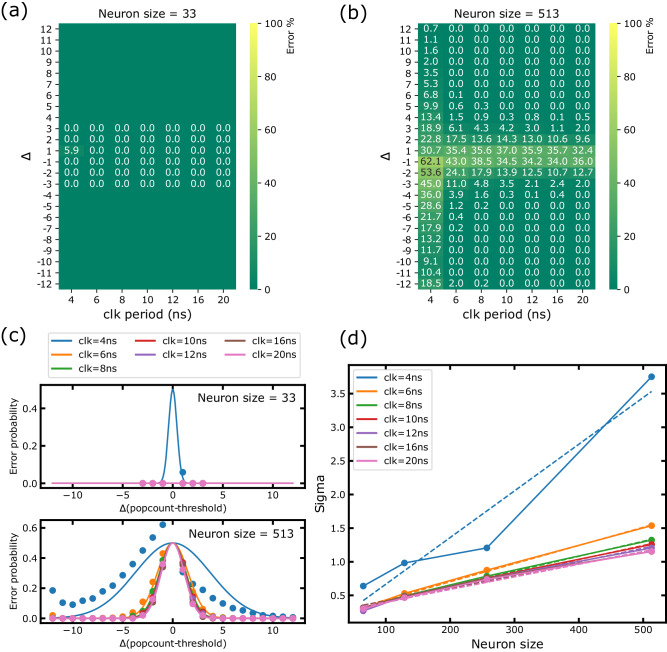
Monte Carlo simulations of neuron operation. Simulated neuron error rate, as a function of the difference between popcount and threshold (Δ) and of the clock period, for neuron sizes of (**a**) 33 inputs and (**b**) 513 inputs (not counting the bias terms). **c** The corresponding error rates are plotted with a Gaussian fit. These simulations include transistor variability using the foundry design kit and the eXclusive OR (XOR) distributions of Fig. 2. **d** Standard deviation of the Gaussian fit of the simulated neuron error rate, for different neuron sizes and clock periods.

Figure 5a–b shows the extracted neuron error distributions for the 33 and 513-inputs neurons, together with a Gaussian fit of the results. Consistently with our measured results, for neurons up to 33 inputs, the output presents no errors for a clock period higher or equal to 6 nanoseconds. The smallest Δ value corresponds to a voltage difference of 34 millivolts for 33 inputs. As we increase the neuron sizes, this smallest voltage difference decreases, down to only 2 millivolts for 513 inputs, and thus, the error rate increases for small Δ values. Fortunately, the Gaussian error distributions remain tight for clock periods higher or equal to 6 nanoseconds.

In the case of 513 inputs, Fig. 5 reveals a high error rate when the difference between popcount and threshold is one. This suggests that the readout circuit presents a precision equivalent to having half the number of attainable voltage levels, resulting in an approximate equivalent precision of 8 bits instead of 9 bits.

Further analysis of the simulation results shows that 93.4% of the neuron errors are due to the comparator, the remainder being due to the clock and clear buffers, and the pull-down clear transistors. Figure 5d shows the obtained standard deviation for other neuron sizes for the different clock periods. As expected, the longer the clock period is, the lower the standard deviation is, as more time is given for the clear and capacitive divider voltage settling. Based on these results, we can set the minimum clock period to 6 nanoseconds.

#### Full BNN error model based on chip characterization and large BNN circuit simulation

We now implement a full neuron error model as a function of neuron size, read voltage, programming compliance current, and clock period, based on the results presented in the last two subsections: XNOR errors are modeled using our experimental results directly, while neuron circuits error is based on the Gaussian fits of our Monte Carlo results. The mathematical details of the model are presented in the “Error model" section in Methods. Based on our error model, we focus here on a large neuron behavior for various usage conditions.

**Fig. 6:**
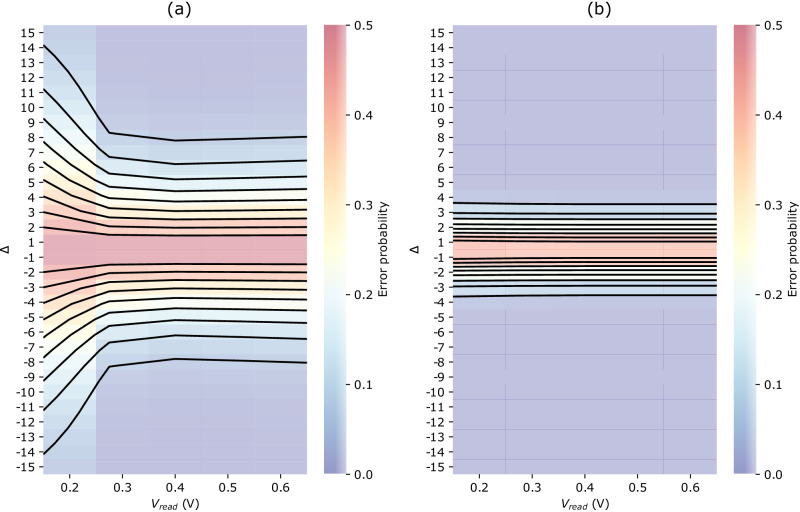
Modeling of a 513-input neurons. Heatmap view of the error probability distributions for a neuron with 513 inputs and a clock period of 6 nanoseconds, as a function of read voltage and Δ value (difference between popcount and threshold value). The iso-lines correspond to error probabilities ranging from 0.05 to 0.5 with a step of 0.05. **a**, **b** correspond to a compliance current I_CC_ of 40 microamperes and 150 microamperes. The error model is detailed in the “Error model” section in Methods.

Figure 6 plots the error probability of a neuron with 513 inputs for a clock period of six nanoseconds, and various compliance currents and read voltages. The read voltage has a very limited impact on the error distribution evolution, especially with a good initial programming current as in Fig. 6b. This observation leads us to choose a low V_read_ to minimize the power consumption during inference without any noticeable impact on the neuron error probability.

### BNN circuit performances at neural network scale

**Fig. 7:**
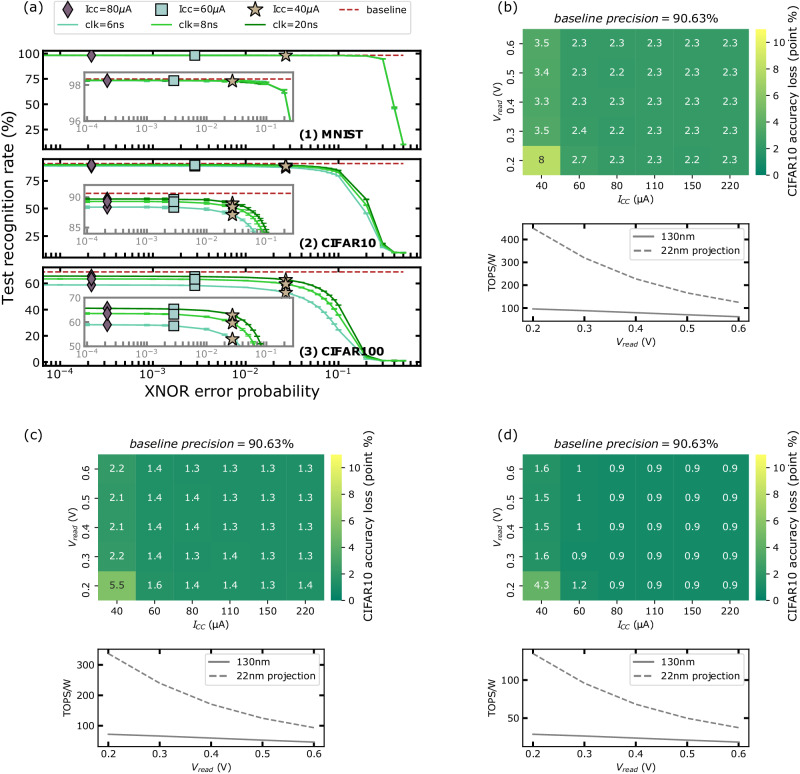
Neural network simulations. **a** Inference accuracy for the MNIST, CIFAR-10 and CIFAR-100 datasets, as a function of eXclusive NOR (XNOR) error probability. Markers indicate the inference precision for I_CC_ = 40 microamperes, 60 microamperes, and 80 microamperes with V_read_ = 0.3 volts. CIFAR-10 accuracy loss (in percentage points, compared to the software precision baseline of 90.6%) for the different compliance current and read voltage V_read_, along with the corresponding energy efficiency in TOPS/W, for a 513-inputs neuron and a clock period of (**b**) 6 nanoseconds, (**c**) 8 nanoseconds and (**d**) 20 nanoseconds (see the “ReRAM simulation model”, “Error model” and “Neural network simulation” sections in Methods for the implementation details).

To evaluate the performance of our BNN circuit at the neural network scale, we incorporated the error model introduced in the previous section (and described in the “Error model” section in Methods - equation eq. (10)) into the PyTorch^21^ deep learning simulation framework. Inferences are performed for multiple programming conditions, read voltages, and clock periods on the MNIST handwritten digit recognition and the CIFAR-10 image recognition datasets (see the “Neural network simulation” section in Methods). Therefore, all MNIST and CIFAR results are simulated, but using experimentally measured distributions of SL voltages and the developed error model calibrated on our testchip. Figure 7 shows the obtained test recognition rate, along with error-free baselines. For the MNIST task, a negligible accuracy degradation is reported for all compliance current values. Even for the most critical configuration (a 6 nanoseconds clock period, a read voltage of 0.2 volts and a compliance current of 40 microamperes) the accuracy degradation is only of 0.2% for a baseline accuracy of 98.3%.

CIFAR-10 image recognition is a much more challenging task. Figure 7b–d shows the accuracy loss, compared to a software precision baseline of 90.6%, for various conditions, along with the corresponding energy efficiency in TOPS/W. The accuracy loss (in percentage points) is low, although it is higher than in the MNIST case. Even for a read voltage of 0.2 volts, and for a standard compliance current (110 microamperes), we observe only 0.9% precision loss for a clock period of 20 nanoseconds (1.4% for a clock period of 8 nanoseconds, and 2.3% for a clock of 6 nanoseconds). Overall, the compliance current has a remarkably low impact on the accuracy: only a truly low value of 40 microamperes substantially degrades the accuracy.

We conducted inference on the CIFAR-100 dataset, which is more intricate. As expected, the software accuracy baseline is lower (68.86%) than for the MNIST and CIFAR-10 datasets. Figure 7a(3) shows the CIFAR-100 accuracy curves as a function of XNOR error rate for different clock periods. Accuracy is less resilient than in the CIFAR-10 task (Fig. 7a(2)). Still, despite the increased complexity of the dataset, we noted only a modest precision loss of 3.25% at a clock period of 20 nanoseconds, with a read voltage of 0.2 volts and a standard compliance current of 110 microamperes. For shorter clock periods of 8 nanoseconds and 6 nanoseconds, the precision loss increases to 5.4% and 10%, respectively.

We now estimate the energy efficiency for our 130-nanometer implementation, for a read voltage of 0.2 volts, a compliance current of 110 microamperes, and a clock period of 6 nanoseconds. The mean current of a single 2T2R complementary bit cell is 1.2 microamperes, leading to a power consumption of 135.7 microwatts for a 513 inputs neuron (including the 10% extra cells for the bias). The use of 2T2R complementary resistive bridges drastically decreases the ReRAM current consumption during the neuron operation, as one of the ReRAM devices is always in high resistance state. The mean power consumed by two inverters, two AND gates and two capacitors (including the clear operation) is 2 microwatts when the XNOR result is unchanged, and 4 microwatts when the XNOR value switches. Our simulations showed an inference XNOR activity factor lower than 25%. Based on the 25% value, the total power consumed by the inverters, AND gates, and capacitors of a 513 inputs neuron is 1.4 milliwatts. The complete neuron power consumption is 1.96 milliwatts after adding the buffers, clear transistors, and sense amplifier power consumption. Considering that we perform two operations (multiplication and accumulation) per neuron input (including the bias) with the thresholding operation at the end, the total number of operations, in a single clock period, is 1127, which corresponds to 0.188 TOPS and an energy efficiency of 96 TOPS/W. Additionally, to evaluate the power consumption of our solution in a 22-nanometer technology, we re-designed and simulated our neuron using a commercial 22-nanometer Fully Depleted Silicon On Insulator (FDSOI) technology design kit and obtained an energy efficiency of 449 TOPS/W.

A low read voltage maximizes the BNN circuit energy efficiency and preserves the inference accuracy. A longer clock period decreases the maximal energy efficiency, as shown in Fig. 7b–d: for a read voltage of 0.2 volts, the mean energy efficiency of our 130-nanometer test chip is 96 TOPS/W for a clock period of 6 nanoseconds, 72 TOPS/W for a clock period of 8 nanoseconds, and 29 TOPS/W for a clock period of 20 nanoseconds. Considering the 22-nanometer projection, these numbers become 449 TOPS/W, 337 TOPS/W, and 135 TOPS/W, respectively.

Tables 2 and 3 compares our work with state-of-the-art approaches, both current-based^16,17,22^ and resistive-divider-based^19^. All of them consider a limited number of inputs per cycle of respectively 4, 9, 16, and 8, to limit the impact of ReRAM variability. Compared to these solutions, we achieve similar or higher energy efficiency. However, although these solutions show high efficiency regarding partial MAC operations, they do not include in their evaluation the sum of all the partial MAC as well as the activation cost, as we propose in our solution. Comparing the MNIST and CIFAR-10 accuracy is challenging as most papers do not provide such results. However, when the accuracy is provided^16,22^, we achieve comparable CIFAR-10 accuracy and accuracy loss, while using only binary weights and activations. Thus, our solution offers comparable or superior energy efficiency than contemporary approaches while maintaining accuracy.

**Table 2:** State-of-the-art comparison table (part 1)

	ISSCC^16^			TCAS-II^19^			ISSCC^17^		
	2020			2021			2021		
Node	22 nm			180 nm			22 nm		
Input bits	1	2	4	1			1	4	8
Weight bits	2	4		1			2	4	8
Inputs on...	BL			WL			WL		
MAC scheme	Ohm’s law			Resistive divider			Ohm’s law^a^		
Accumulation scheme	Peripheral circuitry			Resistive divider			Kirchhoff law		
Latency (ns)	9.8	13.1	18.3	15.98	113.65	214.17	4.9	10.3	14.8
TOPS/W	121.38	45.52	28.93	42.6	35.39	30.26	195.7	47.26	11.91
MAC size	16			9			4		
Partial/total MAC	Partial			Partial			Partial		
Need for digital sum?	Yes			Yes			Yes		
Accuracy (MNIST)	N/A	N/A	N/A	N/A	N/A	N/A	N/A	N/A	N/A
Accuracy (CIFAR10)	N/A	90.18%	N/A	N/A	N/A	N/A	N/A	N/A	N/A
Accuracy degradation (MNIST)	N/A	N/A	N/A	N/A	N/A	N/A	N/A	N/A	N/A
accuracy degradation (CIFAR10)	N/A	0.72%	N/A	N/A	N/A	N/A	N/A	N/A	N/A

^a^(with temporal coding for inputs).

**Table 3:** State-of-the-art comparison table (part 2)

	ISSCC^22^		[our work]	
	2022		2022	
Node	22 nm		130/22 nm	
Input bits	1	8	1	
Weight bits	1	8	1	
Inputs on...	WL		BL	
MAC scheme	Ohm’s law		Resistive divider	
Accumulation scheme	Kirchhoff’s law^a^		Capacitive divider	
Latency (ns)	1.59	14.4	6	20
TOPS/W	416.5^b^	21.6^c^	96 / 449.3^d^	28.8 / 134.8^d^
MAC size	16		513	
Partial/total MAC	Partial		Total	
Need for digital sum?	Yes		No	
Accuracy (MNIST)	N/A	N/A	98.26%^e^	98.26%^e^
Accuracy (CIFAR10)	N/A	91.74%	88.32%^e^	89.71%^e^
Accuracy degradation (MNIST)	N/A	N/A	0.07%^e^	0.07%^e^
accuracy degradation (CIFAR10)	N/A	0.46%	2.31%^e^	0.92%^e^

^a^(with BL discharge).

^b^(1286,4 TOPS/W with a sparsity of 90%).

^c^(61.8 TOPS/W with a sparsity of 90%).

^d^(22 nm typical simulation).

^e^(V_read_ = 0.2 V and I_CC_ = 110 μA).

The most natural baseline for the neuron circuit would be a fully digital implementation, which would show no computation error and thus achieve software baseline accuracy, but at the cost of a higher power consumption. We have not performed the energy consumption study, but it has been thoroughly been performed in the initial work that inspired us^7^ (in this case, the neuron circuit is used with SRAM). In their work, they estimated the energy per classification of the switch capacitor array to be 4.2 times lower when compared to a hand-designed digital implementation of the switch capacitor neuron. In the digital implementation, a digital Wallace tree adder is used instead of the analog capacitive DAC circuit.

Note that, although truly state-of-the-art performance on more complex datasets such as CIFAR-100 and ImageNet requires multi-bits weights and neuronal activations, recent research has shown surprisingly high performance on these datasets using binarized neural networks. For example, using a dedicated type of neural architecture search^23^ achieved 66.5% TOP-1 and 86.8% TOP-5 accuracy on ImageNet using fully binarized neural networks. These results are promising for the development of binary neuromorphic circuits implementing relatively complex tasks.

## Conclusions

In this work, we characterize for the first time a BNN circuit based on a 2T2R RRAM array with a capacitive output neuron and demonstrate experimentally its high robustness against ReRAM variability. The XNOR values are computed in memory using 2T2R ReRAM cells with complementary weight coding and a single inverter located at the bottom of the source line. The popcount and threshold operations are implemented with a fully differential capacitive divider, which naturally presents low variability, allowing the realization of large ReRAM-based neurons (with up to 513 inputs). Measurement shows very good performances with low XNOR and neuron error rates. The neuron robustness is studied with different programming conditions, ReRAM operating voltages, and clock periods, and a neuron error model is developed and tuned on the measured and simulated circuit responses, before being embedded in the Pytorch environment to perform BNN inferences on the MNIST and CIFAR-10 datasets. These neural network simulations reveal that due to the intrinsic tolerance of binarized neural networks to errors, it can be favorable to choose low read voltages and programming currents, as they respectively promote energy efficiency and device endurance, with low impact on network-level accuracy. For a clock period of 6 nanoseconds, our 513 inputs BNN circuit provides an appealing peak energy efficiency of 96 TOPS/W and 449.3 TOPS/W for respectively a 130-nanometer and a 22-nanometer implementation.

## Methods

### Test chips fabrication

Most experimental results of the paper were obtained using the test chip presented in Fig. 1. This test chip implements the proposed BNN circuit and is designed and fabricated in a 130 nm CMOS technology with co-integrated ReRAM cells in the back-end-of-line between metal layers M4 and M5. ReRAM cells are composed of a HfO2 layer sandwiched between TiN and Ti/TiN layers. Figure 4 plots the V_SL_ voltage, which is not readily available in the BNN test chip. For this purpose, we use a different test chip, constituted of a simple 1204 2T2R ReRAM array. The ReRAM cells of this test chip are using the same materials as the BNN chip, and were fabricated in an earlier run. In our fabricated circuit, we used 105 fF capacitors. This choice of relatively large capacitors was a conservatice choice to ensure circuit functionality. On the other hand, in the simulations used to estimate the energy consumption of scaled-up systems, we used a more aggressive value of 3.9fF.

### ReRAM simulation model

We used a ReRAM Verilog-A model to design the programming circuits of our demonstrator. For simulation of the neuron circuits, we preferred to use another methodology to capture accurately the impact of the variability of ReRAM (which is very challenging to model in a Verilog-A model): we use experimentally-measured SL voltage distributions for the XOR operation (measured on the 130-nm fabricated demonstrator) as inputs of the simulations. For the 22 nm FDSOI technology, to stay on the side of caution, we assumed that the thick-oxide access transistors used in the 22 nm node would be equivalent to those used in the 130 nm node within the ReRAM array, and we used the same SL voltage distributions.

### 2T2R programming

**Fig. 8:**
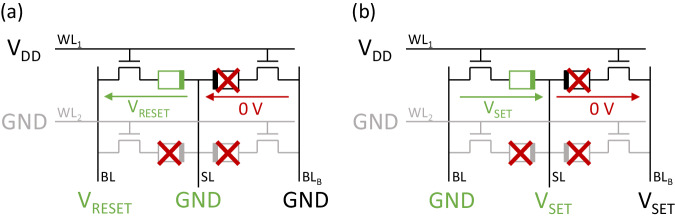
2-Transistors-2-Resistors (2T2R) SET and RESET schemes. **a** SET and (**b**) RESET operations on a 2T2R bitcell. SL Source Line, BL Bit Line, WL Word Line.

Figure 8 illustrates the biasing conditions to program a specific ReRAM in a 2T2R bitcell. The device to be programmed is highlighted in green in Fig. 8. The programming process begins with the activation of the selected word-line (WL). The RESET or SET voltages are then applied across the selected device through the BL and SL. Since the second ReRAM device in the selected 2T2R cell is also acceded due to the shared WL, an inhibition voltage needs to be applied to avoid any write disturb. Therefore, in the case of a RESET operation, the BL of the unselected ReRAM device is grounded (Fig. 8a), whereas for a SET operation it is biased at the SET voltage (Fig. 8b). The total writing time of a single ReRAM device is 2 microseconds. No write-and-verify technics have been used.

### IR drop projection on large memory array

Our approach is specifically designed to effectively address IR drop issues by implementing a single WL activation at a time. This method ensures a limited current of 1.2 microamperes in each 2T2R cell per column, calculated as V_read_/(R_HRS_ + R_LRS_), assuming a compliance current of 110 microamperes and a V_read_ of 0.2 volts. To demonstrate the resilience of our XNOR operations against IR drop, we conducted an analysis considering the parasitic resistors of the metal lines for each bitcell. We calculated the V_SL_ voltage, accounting for the IR drop, with a V_read_ of 0.2 volts, for column size up to 1kbits. For the top row, the V_SL_ voltage reaches a high value of 0.69V or a low value of 0.51V. At the bottom row (1000th row), the (V_SL_) voltage is reduced by 1.35% for the high value and respectively 1.83% for the low value. This very limited reduction in the V_SL_ voltage swing, from the top to the last row, demonstrates the robustness of our approach when applied to large memory array sizes.

### Read-disturb experimental evaluation

**Fig. 9:**
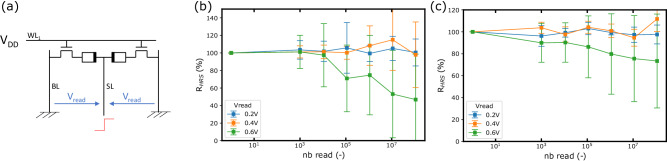
Read-disturb measurements. The read cycle experiment is performed on cells programmed in a complementary manner with the following conditions: SET operation - V_SET_ = 2 volts with a current compliance of I_CC_ = 200 microamperes, SET duration t_SET_ = 1 microseconds. RESET operation - V_RESET_ = 2.5 volts without any current limitation, RESET duration t_RESET_ = 10 microseconds. **a** 2-Transistors-2-Resistors (2T2R) cycling setup, normalized cycling measurement on R_HRS_ without (**b**) and with (**c**) 1ms relaxation. SL Source Line, BL Bit Line, WL Word Line.

The primary motivation behind selecting the lowest possible voltage for ReRAM devices during MAC (Multiply and Accumulate) operations is to minimize inference power consumption. Operating at low voltages also serves as an effective strategy to mitigate read disturb issues. In our specific case, the voltage V_read_ across the bit lines is shared between two complementary ReRAM devices connected in series. As a result, each device experiences a voltage lower than V_read_, and the HRS cell always experiences a greater voltage drop compared to the low resistance state (LRS) cell, making it more prone to read disturb issues. We conducted read cycling experiments on 2T2R ReRAM cells programmed in a complementary manner, to evaluate read disturb effects. For the read cycling experiments, V_read_ voltages ranging from 0.2 volts to 0.6 volts are applied. Both BL (bit line) and BL_B_ (complementary bit line) are grounded, and the V_read_ voltage is directly applied to the selected source line (SL). The V_read_ voltages are delivered as successive pulses with rise and fall times of 100 nanoseconds and an application duration of 100 nanoseconds. Within each decade, we extract the high resistance state (HRS) value with a pulse of 0.1 volts for a duration of 10 microseconds. In these experiments, the full V_read_ voltage is applied on each ReRAM, and timings are above the one used in our design, due to generator limitation: these cycling experiments represent a worst-case scenario both in terms of voltage and timing. The measured HRS resistance for various V_read_ pulses up to 10^8^ cycles is shown in Fig. 9b. For V_read_ voltages of 0.4 volts and below, no discernible trend of read disturb is observed in terms of mean HRS values, even after 10^8^ cycles. The only noticeable change is a larger dispersion of the HRS values compared to the initial distribution. Conversely, we observe a clear disturbance when V_read_ reaches 0.6 volts, for pulse counts exceeding 10^5^, resulting in the HRS value being reduced to 70% of its original value. Figure 9c presents the same measurements with an added one-millisecond relaxation time between each pulse. We observe a clear reduction in read disturb: for V_read_ = 0.6 V, the HRS value decreases to only 86% of its original value after 10^5^ cycles. Again, for V_read_ values equal to or below 0.4 volts, no definitive trend of read disturb on the mean HRS values is observed, although there is a larger dispersion of the HRS values compared to the initial distribution. Applying pulses with a duration of 100 nanoseconds represents an extreme case as we considered clock periods below 20 nanoseconds in our design. If we assume that a 100 nanoseconds pulse is equivalent to 100 pulses of one nanosecond, we can extrapolate that our design would be read disturb free for a read voltage of 0.2 volts, even after 10^10^ cycles, considering a worst case scenario.

### In-Memory XNOR operation

The weight coding is the following: (R,R_B_) = (HRS,LRS) if *w*_*ij*_ = 1, and (R,R_B_) = (LRS,HRS) if *w*_*ij*_ = −1, with LRS being the Low Resistance and HRS the High Resistance State of the ReRAM. The activation input is applied on the Bit Lines (BL) and complementary bit lines (BL_B_) also in a complementary fashion, such as $({{{{{{{{\rm{V}}}}}}}}}_{{{{{{{{\rm{BL}}}}}}}}},{{{{{{{{\rm{V}}}}}}}}}_{{{{{{{{{\rm{BL}}}}}}}}}_{{{{{{{{\rm{B}}}}}}}}}})$=(V_HIGH_,V_LOW_) if *in*_*i*_ = 1, and $({{{{{{{{\rm{V}}}}}}}}}_{{{{{{{{\rm{BL}}}}}}}}},{{{{{{{{\rm{V}}}}}}}}}_{{{{{{{{{\rm{BL}}}}}}}}}_{{{{{{{{\rm{B}}}}}}}}}})$=(V_LOW_,V_HIGH_) if *in*_*i*_ = −1. Once the activation input is applied, the 2T2R structure behaves as a resistive bridge, whose middle point, the Source Line (SL) voltage, is given by

5 $${V}_{{{{{{{{\rm{SL}}}}}}}}}= 	 \, ({V}_{{{{{{{{\rm{BL}}}}}}}}}-{V}_{{{{{{{{{\rm{BL}}}}}}}}}_{{{{{{{{\rm{B}}}}}}}}}}).\frac{{R}_{{{{{{{{\rm{B}}}}}}}}}}{(R+{R}_{{{{{{{{\rm{B}}}}}}}}})}+{V}_{{{{{{{{{\rm{BL}}}}}}}}}_{{{{{{{{\rm{B}}}}}}}}}}\,\,when\,{V}_{{{{{{{{\rm{BL}}}}}}}}} \, > \, {V}_{{{{{{{{{\rm{BL}}}}}}}}}_{{{{{{{{\rm{B}}}}}}}}}}\,(i{n}_{i}=1)\\ {V}_{{{{{{{{\rm{SL}}}}}}}}}= 	 \, ({V}_{{{{{{{{{\rm{BL}}}}}}}}}_{{{{{{{{\rm{B}}}}}}}}}}-{V}_{{{{{{{{\rm{BL}}}}}}}}}).\frac{R}{(R+{R}_{{{{{{{{\rm{B}}}}}}}}})}+{V}_{{{{{{{{\rm{BL}}}}}}}}}\,\,when\,{V}_{{{{{{{{\rm{BL}}}}}}}}} \, < \, {V}_{{{{{{{{{\rm{BL}}}}}}}}}_{{{{{{{{\rm{B}}}}}}}}}}\,(i{n}_{i}=-1).$$

We choose V_LOW_ and V_HIGH_ to be symmetric with respect to V_DD_/2 (V_DD_ being the circuit supply voltage). The voltage V_read_ = V_HIGH_-V_LOW_ serves as the read voltage for the devices and is chosen lower than the ReRAM threshold voltage to avoid read-disturbance during the XNOR operation. As the ReRAMs are coded to complementary values, the ReRAM in the HRS state always takes the largest voltage drop and pushes V_SL_ to the bit line voltage of the ReRAM in the LRS state. Consequently, by eq. (5), the V_SL_ voltage follows the XOR truth table (see Table 1).

**Fig. 10:**
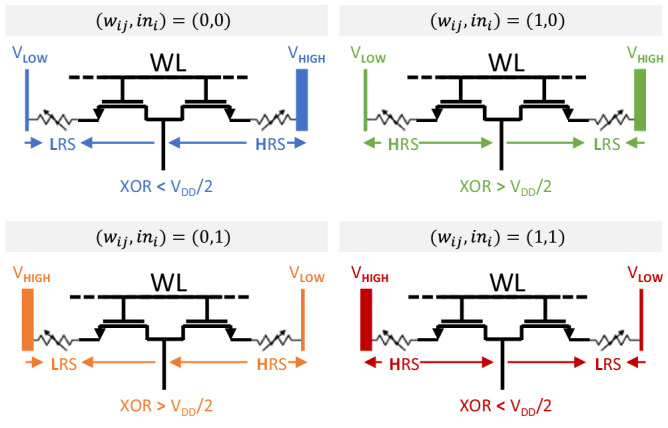
EXclusive OR (XOR) operating scheme for the four cases of the truth table. V_LOW_ and V_HIGH_ are chosen to be symmetric with respect to V_DD_/2. With ReRAMs programmed as complementary values, the HRS cell consistently experiences the most significant voltage drop, effectively pushing V_SL_ to the bit line voltage of the LRS cell. We can see that the V_SL_ voltage follows the XOR truth table. WL Word Line, HRS High Resistive State, LRS Low Resistive State.

Figure 10 illustrates the operating principle of the XOR gate. The SL voltage is naturally pulled towards the voltage of the LRS (low resistance state) of the 2T2R cell thanks to the resistive bridge structure.

**Fig. 11:**
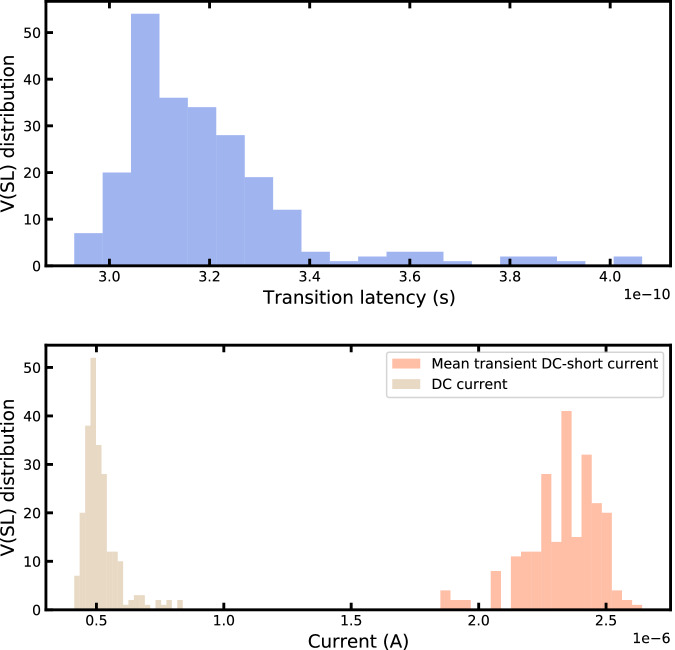
Inverter latency and current simulations. Inverter transition latency, mean transient Direct Current (DC)-short current and DC current for a compliance current of I_CC_ = 110 microamperes and V_read_ = 0.2 volts.

We can notice that the voltage on SL does not fully swing, which has an impact on the transition latency and transient DC-short current, as illustrated in Fig. 11. We performed transient simulations on our inverter structure and extracted its transition latency and mean DC transient DC-short current. V_SL_ voltages are raised from 0 volts to their final value with a rising time of 300 picoseconds. This is realistic as before the WL are activated and the BL/BL_B_ voltages are applied to perform the XNOR operation, the SL voltage is equal to zero, and this voltage raises to V_DD_/2 ± V_read_/2 once the WL are activated. The transition latency is computed as the latency between the time where the V_SL_ signal reaches 50% of its total dynamic and the time when the inverter’s output reaches 50% of its total dynamic. The transient DC-short current is computed as the mean current while the inverter output falls from 90% to 10% of its output dynamic. Using a compliance current of I_CC_ = 110 microamperes and V_read_ = 0.2 volts, we obtain a transition latency ranging between [0.29, 0.41] nanoseconds, a mean transient DC-short current ranging between [1.85, 2.64] microamperes, and a DC current ranging between [0.41, 0.84] microamperes. Compared to a full swing SL input, with a V_SL_ voltage raised to V_DD_ = 1.2 volts, the mean transition latency is increased by 0.24 nanoseconds, the mean transient DC-short current is reduced by 16.9 microamperes as the inverter’s peak of current is lower for low V_SL_ swings, and the mean DC current is increased by 0.52 microamperes. The obtained mean transient current values are low due to the high threshold voltage technology used in our design.

### Threshold adjustment in the near-memory popcount operation

**Fig. 12:**
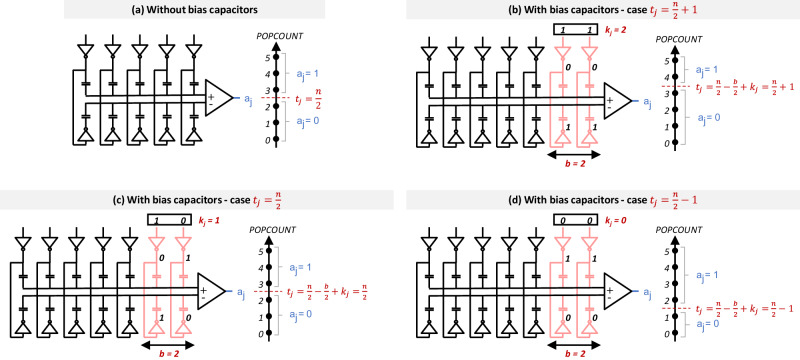
Method for adjusting neuron threshold. **a** Capacitive divider structure without additional bias capacitors for a five-inputs neuron: the threshold *t*_*j*_ is $\frac{n}{2}$. Same capacitive divider structure with two additional bias capacitors, in the case of (**b**) ${t}_{j}=\frac{n}{2}+1$, (**c**) ${t}_{j}=\frac{n}{2}$ and (**d**) ${t}_{j}=\frac{n}{2}-1$.

As illustrated in Fig. 12b–d, depending on the number *k*_*j*_ of ones applied on the SLs of the bias columns (through the same resistive bridge approach as for the XNOR operators), the popcount capacitive bridge will be advantaged or disadvantaged compared to the second capacitive bridge, thus shifting the threshold value *t*_*j*_ down to a minimal value of ${t}_{{j}_{\min }}=\frac{n}{2}-\frac{b}{2}$ (*k*_*j*_ = *b*) or up to a maximal value of ${t}_{{j}_{\max }}=\frac{n}{2}+\frac{b}{2}$ (*k*_*j*_ = 0). The voltage of the two capacitive dividers is then given by

6 $${V}_{{{{{{{{\rm{PC}}}}}}}}}= 	 \frac{m+(b-{k}_{j})}{n+b}.{V}_{{{{{{{{\rm{DD}}}}}}}}}\\ {V}_{{{{{{{{\rm{PCB}}}}}}}}}= 	 {V}_{{{{{{{{\rm{DD}}}}}}}}}-\frac{m+(b-{k}_{j})}{n+b}.{V}_{{{{{{{{\rm{DD}}}}}}}}},$$

and the comparator output *a*_*j*_ by

7 $${a}_{j} = 	 \, {{{{{{{\rm{sign}}}}}}}}({V}_{{{{{{{{\rm{PCB}}}}}}}}}-({V}_{{{{{{{{\rm{DD}}}}}}}}}-{V}_{{{{{{{{\rm{PCB}}}}}}}}}))= {{{{{{{\rm{sign}}}}}}}}\left(m-\left(\frac{n}{2}-\frac{b}{2}+{k}_{j}\right)\right)\\ = 	 \, {{{{{{{\rm{sign}}}}}}}}(m-{t}_{j}),$$

which corresponds to eq. (2), *m* being the popcount value. Therefore, the threshold *t*_*j*_ is

8 $${t}_{j}=\frac{n}{2}-\frac{b}{2}+{k}_{j},$$

with *k*_*j*_ an integer in the range 0 to *b*.

### Test chip characterization

To program the ReRAM cells we use standard RESET conditions in all situations (*t*_RESET_ = 1 *microseconds*, *V*_WL_(RESET) = 4 *volts*, *V*_RESET_ = 2 *volts*). Only the SET conditions differ, with compliance currents I_CC_ ranging from 40 to 220 microamperes and *t*_SET_ = 1 *microseconds*, *V*_WL_(SET) = 2 *volts*, *V*_SET_ ∈ [1.1, 2] *volts*. To extract the error probabilities in Fig. 3, we wrote 230 synaptic weights and measured their respective XNOR and neuron output values. We repeated this experiment for all combinations of voltages V_read_ ranging from 0.2 to 0.6 volts, and compliance current going from 40 to 220 microamperes, and Δ – the difference between the neuron’s two capacitive dividers inputs set to one – going from −3 to 3. For each condition, we repeated the write and measurements steps ten times. Thus, for each V_read_ and I_CC_ conditions, the XNOR error probability is based on 13,800 measurements. Similarly, for each V_read_, I_CC_ and Δ values, the neuron error probability is based on 100 measurements.

### On-chip operation of the popcount computation

To clear the capacitors before each operation, we included AND gates between the inverters’ outputs and the capacitors, along with pull-down transistors connected to the two capacitive dividers. The typical neuron operation during an inference step is as follows. After selecting a given word line WL, the activation values of the selected set of input neurons are applied to the bit line BL, to generate the XNOR values. In parallel, the clear (CLR) signal is activated to ground the top and bottom electrodes of the neuron capacitors, to remove the charge. The XNOR digital outputs are prevented from reaching the capacitors by the AND gates and the clear signal set to V_DD_. When the clear phase ends, the XNOR values pass through the AND gates to reach the capacitors, settling the popcount/threshold voltages. The comparator compares the two capacitive divider voltages and computes the output neuron activation. This full operation takes one clock cycle.

### Error model

We consider a neuron *a*_*j*_ with N inputs (including the bias terms), n_1_ of each are expected to lead to a one XNOR value. For simplicity, we focus on the case ${n}_{1}\le \left\lfloor N/2\right\rfloor$, so that *a*_*j*_ is expected to be one (our derivation can be adapted to the other case straightforwardly). We call *p* the probability for a single 2T2R-based XNOR operator to give an erroneous output, which we extract for various programming conditions and read voltages from the experimental measurements of Fig. 3. We can obtain P({f_0_ = i}) the probability of having i XNOR outputs turning from a correct zero state to an erroneous one state, and P({f_1_ = j}) the probability of having j XNOR outputs turning from a correct one state to an erroneous zero state, using binomial laws

9 $$\begin{array}{rcl}P(\{{f}_{0}=i\})&=&\left(\begin{array}{c}N-{n}_{1}\\ i\end{array}\right)\times {p}^{i}\times {(1-p)}^{N-{n}_{1}-i}\\ P(\{{f}_{1}=j\})&=&\left(\begin{array}{c}{n}_{1}\\ j\end{array}\right)\times {p}^{\, j}\times {(1-p)}^{{n}_{1}-j}.\end{array}$$

We also introduce P({CN(x) = 1}) the probability of the capacitive neuron (CN) giving an output of one when x XNOR outputs equal to one, obtained for various neuron sizes and clock periods from the Gaussian distributions of Fig. 5d. Then, we can compute the probability of the neuron output *a*_*j*_ being equal to one instead of zero by

10 $$ 	 P(\{{a}_{j}= 1| {n}_{1}\le \left\lfloor N/2\right\rfloor \})\\ = 	 {\sum}_{i=\lceil N/2\rceil -{n}_{1}}^{N-{n}_{1}}P(\{{f}_{0}=i\}){\sum}_{j=0}^{\min ({n}_{1},{n}_{1}+i-\lceil N/2\rceil )} \\ 	 \quad P(\{{f}_{1}=j\}) \times P(\{{{{{{{{\rm{CN}}}}}}}}({n}_{1}+i-j)=1\})\left.\right)\\ 	 \quad + {\sum}_{i=0}^{\left\lfloor N/2\right\rfloor }P(\{{f}_{0}=i\}){\sum}_{j=\max (0,{n}_{1}+i-\left\lfloor N/2\right\rfloor )}^{{n}_{1}} \\ 	 \quad P(\{{f}_{1}=j\})\times P(\{{{{{{{{\rm{CN}}}}}}}}({n}_{1}+i-j)=1\})$$

### Impact of the proposed approach on the neuron error reduction

**Fig. 13:**
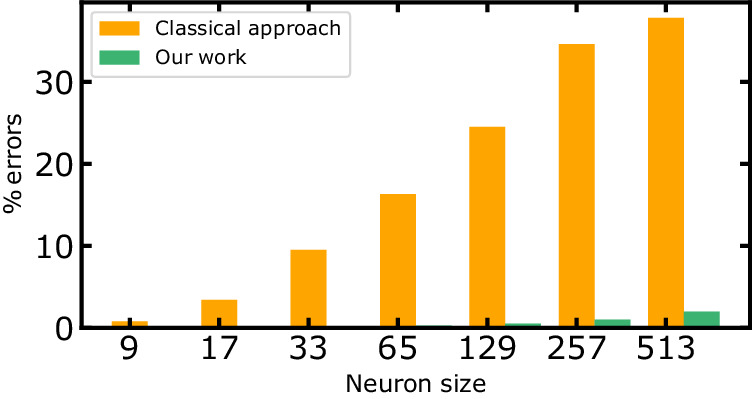
Neuron maximal error percentage due to Resistive Random Access Memories (ReRAMs) for I_CC_ = 110 μA. Our approach demonstrates an important reduction in neuron error rates caused by ReRAM variability compared to classical analog in-memory computing.

To assess the robustness of our approach taking into account ReRAM variability, we compared it to a 1T1R fully analog-in-memory computing approach, using Ohm and Kirchhoff’s law for MAC operations. Figure 13 presents a comparison of neuron error rates attributed to ReRAMs in our approach (validated through experiments) and the expected error rates due to ReRAMs using analog in-memory computing with the same ReRAM variability. The V_read_ voltage is set at 0.3 V, and the compliance current is 110 μA to align with our work’s conditions. This Figure underscores that our approach largely mitigates the impact of ReRAM variability, even under these challenging conditions. This Figure is based on the variability our hafnium oxide device technology, and the exact benefits of our approach could vary depending on electrical properties of memory devices and network architectures choices.

### Neural network simulation

Errors are not considered for the first and last layers, since they are not binarized. For the MNIST task, we used a fully connected network with three hidden layers of 1025 neurons each. For the more challenging CIFAR-10 task, we used a binarized Visual Geometry Group (VGG) structure^24^, consisting of six convolutional layers followed by three fully connected layers^2^.

## Data Availability

Data measured in this study is available from the corresponding authors upon request. The datasets used to evaluate the neural networks are available publicly online. The MNIST database of handwritten digits is available on http://yann.lecun.org/exdb/mnist/index.html. The CIFAR-10 and CIFAR-100 datasets are available on https://www.cs.toronto.edu/~kriz/cifar.html.

## Code availability

The software programs used for modeling the Binarized Neural Network are available from the corresponding authors upon request.
